# Variation within and between Closely Related Species Uncovers High Intra-Specific Variability in Dispersal

**DOI:** 10.1371/journal.pone.0011123

**Published:** 2010-06-15

**Authors:** Virginie M. Stevens, Sandrine Pavoine, Michel Baguette

**Affiliations:** 1 F.R.S.-FNRS and Université de Liège, Unité de biologie du comportement, Liège, Belgium; 2 UMR CNRS-MNHN 7179, Muséum National d'Histoire Naturelle, Brunoy, France; 3 UMR CNRS-MNHN 7204, Muséum National d'Histoire Naturelle, Département d'Ecologie et Gestion de la biodiversité, Paris, France; 4 USR CNRS 2936 Station d'Ecologie Expérimentale du CNRS à Moulis, Saint-Girons, France; Texas A&M University, United States of America

## Abstract

Mounting evidence shows that contrasting selection pressures generate variability in dispersal patterns among individuals or populations of the same species, with potential impacts on both species dynamics and evolution. However, this variability is hardly considered in empirical works, where a single dispersal function is considered to adequately reflect the species-specific dispersal ability, suggesting thereby that within-species variation is negligible as regard to inter-specific differences in dispersal abilities. We propose here an original method to make the comparison of intra- and inter-specific variability in dispersal, by decomposing the diversity of that trait along a phylogeny of closely related species. We used as test group European butterflies that are classic study organisms in spatial ecology. We apply the analysis separately to eight metrics that reflect the dispersal propensity, the dispersal ability or the dispersal efficiency of populations and species. At the inter-specific level, only the dispersal ability showed the signature of a phylogenetic signal while neither the dispersal propensity nor the dispersal efficiency did. At the within-species level, the partitioning of dispersal diversity showed that dispersal was variable or highly variable among populations: intra-specific variability represented from 11% to 133% of inter-specific variability in dispersal metrics. This finding shows that dispersal variation is far from negligible in the wild. Understanding the processes behind this high within-species variation should allow us to properly account for dispersal in demographic models. Accordingly, to encompass the within species variability in life histories the use of more than one value per trait per species should be encouraged in the construction of databases aiming at being sources for modelling purposes.

## Introduction

In most mobile animals, locomotory and navigation limits generate broad, evident differences in dispersal patterns of organisms belonging to contrasted clades. Such huge inter-specific differences in the ability to move among local habitat patches are probably the main reason why dispersal has been so long considered as a species-specific fixed trait. However, there is now mounting evidence that dispersal is variable at the species level because populations and individuals may experience contrasting pressures on their dispersal [1], [2].

Theory predicts, and empirical work confirms, that dispersal is condition-dependent [2], [3]. Many environmental factors contribute to the fine tuning of costs and benefits of dispersal, and hence impact the evolution of dispersal in populations. Dispersal is also phenotype-dependent [1]. The fitness expectations at a particular place can be different for individuals that belong to different categories of sex, age, phenotype, developmental conditions, etc. Accordingly, the costs and benefits of dispersal should also vary among individuals within a given population.

**Figure 1:**
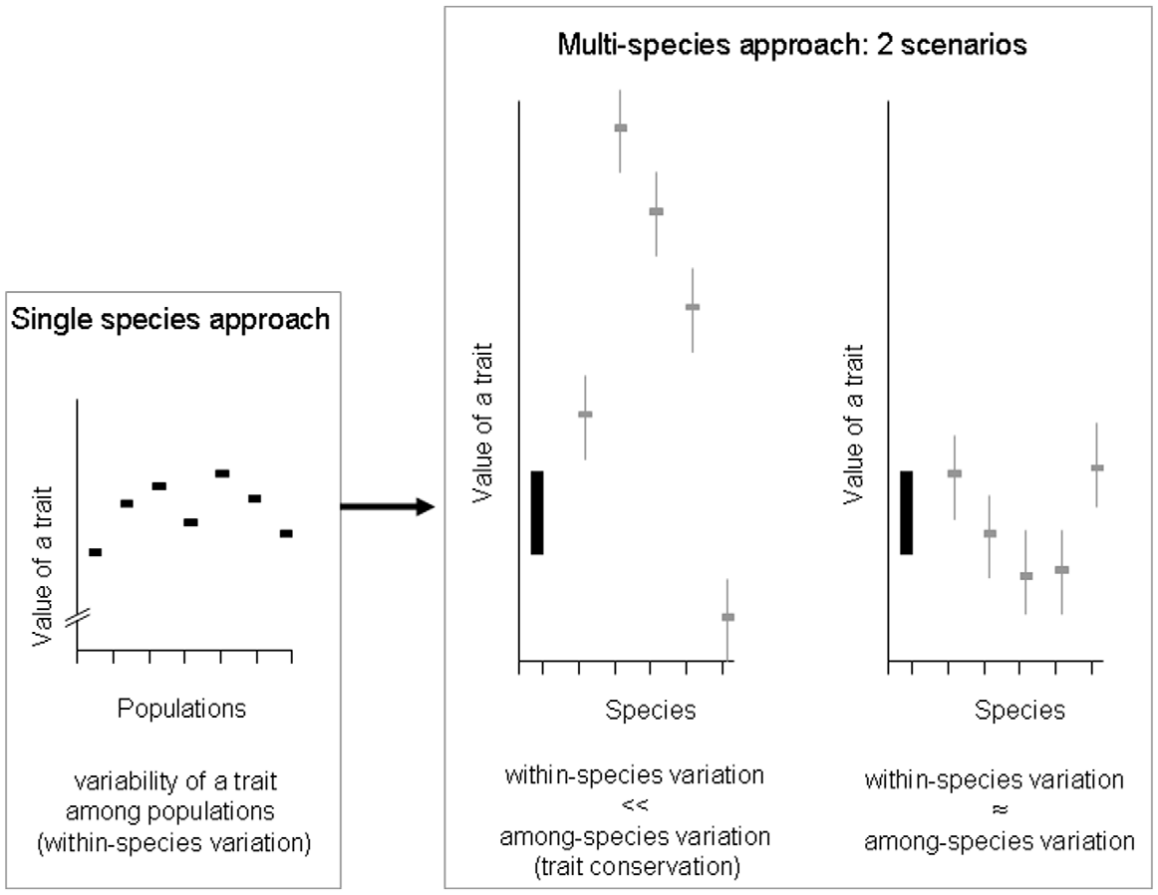
Illustration of the comparison of variation in a trait at intra-specific and inter-specific levels. Small panel: illustration of hypothetical within-species trait variability (among population differences in the value of a trait) considered without inter-specific reference. The large panel illustrate two hypothetical scenarios, where the variability among populations for the species of interest (summarized by the black rectangle) is now viewed in the light of existing inter-specific variation in the trait (grey symbols: trait values in five other species): left, the situation of trait conservation, where intra-specific variability is low relatively to inter-specific variability; right, a situation of high within-species variability, where differences between populations of a species are of the same order of magnitude than among-species differences.

A high intra-specific variation in dispersal ability resulting from both condition- and phenotype-dependence of dispersal costs and benefits is now widely accepted [1], [2], [3]. Although this variation is the core of theoretical studies addressing the evolution of dispersal [4], it is still scarcely documented and considered in empirical works, with the noticeable exception of sex-biased and density-dependent dispersal patterns [5], [6], [7]. For instance, metapopulation models that are commonly used for conservation issues typically assume dispersal to be a fixed function for the species considered (see e.g. [8], [9]). The implicit assumption is that the within-species variation in dispersal is negligible as regard to the variation that exists among species, and that it can therefore be ignored. The intra-specific variation in dispersal was, to the best of our knowledge, never considered in comparison to the variation at the inter-specific level. However, only this comparison can inform us on the relative importance of within-species variation in dispersal traits (Figure 1) and on the legitimacy of ignoring it. Here we used published data of dispersal in European butterflies to fill this gap and make the comparison in a phylogenetic context.

Butterflies have long been recognized as ideal models for the study of fragmented populations and have now been widely adopted as biological models in the integrated study of dispersal [10], [11], [12]. Using published dispersal performances of butterflies, we investigated (i) if there is a phylogenetic signal in the diversity of dispersal traits in butterflies, and (ii) how this signal partitions onto the phylogenetic tree of this highly diverse taxonomic group.

The relative amount of diversity for a given trait (here dispersal) that is supported by ancient nodes and close-to-tips nodes of a phylogenetic tree provides us with information about the evolutionary history of that trait. By partitioning the functional diversity, it is possible to contrast situations in which the trait evolved early, and then was conserved (in that case, the diversity in trait values would tend to be rooted into the tree) and situations where the trait evolved recently (in that case, closely related species would show different values for the trait and diversity would be skewed to close-to-tip nodes of the tree) [13].

Here, we partitioned the diversity in dispersal traits to assess the importance of the intra-specific diversity in dispersal relative to the diversity observed across species. We considered the values of eight dispersal metrics assessed in different populations of a species as the source of within-species variation and ignored the part of the variation attributable to differences among individuals of the same population. To make the comparison in a phylogenetic context, we considered populations of a given species such as these were distinct sister-taxa (virtual taxa), and hence were supported by the closest-to-tips nodes of the phylogenetic tree. If the intra-specific variation in dispersal ability of butterfly is less than the amount of variation expressed at the inter-specific level, we expect that these terminal nodes, supporting populations of a given species, will also support a significantly lower part of the diversity than other nodes.

## Results

### Phylogenetic signal

Ignoring the within-species variation in dispersal (that is, using values of each metric averaged over populations of each species), we found that there are significant phylogenetic signals in two dispersal metrics coming from multisite mark-recapture studies (*alpha2* and *daily moves*), but not in the genetic structuring among populations at any of the three scales considered (*FstL*, *FstR*, and *FstC*; Table 1).

**Table 1:** Test for a phylogenetic signal in dispersal for European butterflies.

Trait	Metric^a^	Number of species	Abouheif's *C_mean_*	*P*>*C_mean_* b
*Dispersal propensity*	*Dispersal fraction* c	25	0.233	0.208
*Dispersal ability*	*Alpha1* d	16	0.258	0.208
	*Alpha2* e	18	0.542	0.008
	*P5Km* d	27	0.263	0.132
	*Daily moves* f	25	0.374	0.070
*Dispersal efficiency*	*FstL* g	13	0.144	0.514
	*FstR*	15	0.063	0.640
	*FstC*	10	0.125	0.514

a The value of the dispersal metric considered is the mean value observed across replicates (where applicable).

b *P* are adjusted *P*-values.

c *Dispersal fraction*: proportion of recaptures with inter-patch movement in multisite mark-recapture.

d *Alpha1* and *alpha2*: descriptors of the shape of a negative exponential dispersal kernel measured in small (<1.9 km) or large (>1.9 km) study sites.

e *P5km*: probability of dispersal movement ≥5 km, estimated from the shape of inverse power dispersal kernels.

f *Daily moves*: mean daily displacements in multisite mark-recapture.

g *FstL*, *FstR* and *FstC*: measure of genetic structuring (*F*_ST_) from allozyme surveys respectively at the landscape scale (<100 km), the regional scale (100-600 km), or the continental scale (>600 km).

### Decomposition of dispersal diversity

**Figure 2:**
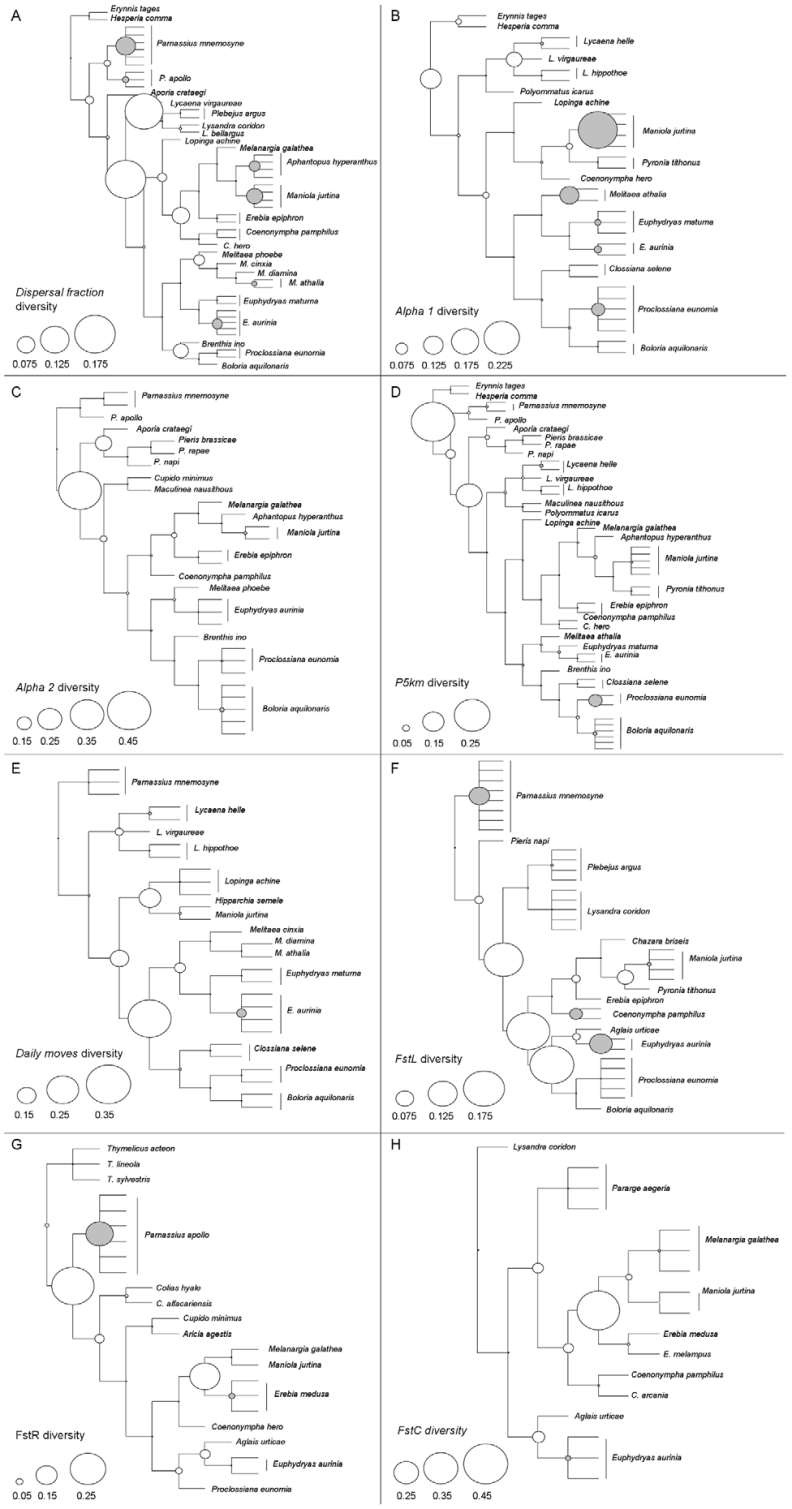
Decomposition of dispersal diversity along the butterfly phylogeny. The circles at nodes provide the contribution of the node to total diversity in dispersal metric. The scale is given at the bottom left-hand corner of each panel. White circles are for nodes in the original classification, grey circles are for the contribution of within-species diversity to the total diversity. Grey branches denote replicates for a given species, here described as virtual sister-taxa. A: *dispersal fraction*: proportion of recaptures with inter-patch movement in multisite mark-recapture. B, C: respectively *alpha1* and *alpha2* that describe the shape of a negative exponential dispersal kernel measured in small (<1.9 km) or large (>1.9 km) study sites. D: *P5km*, the probability of dispersal movement ≥5 km, estimated from the shape of inverse power dispersal kernels. E: *Daily moves*, the mean daily displacements in mark-release-recapture surveys. F, G, H: *FstL*, *FstR* and *FstC*, measures of the genetic structuring (*F*_ST_) from allozyme surveys respectively at the landscape scale (<100 km), the regional scale (100-600 km), or the continental scale (>600 km).

**Figure 3:**
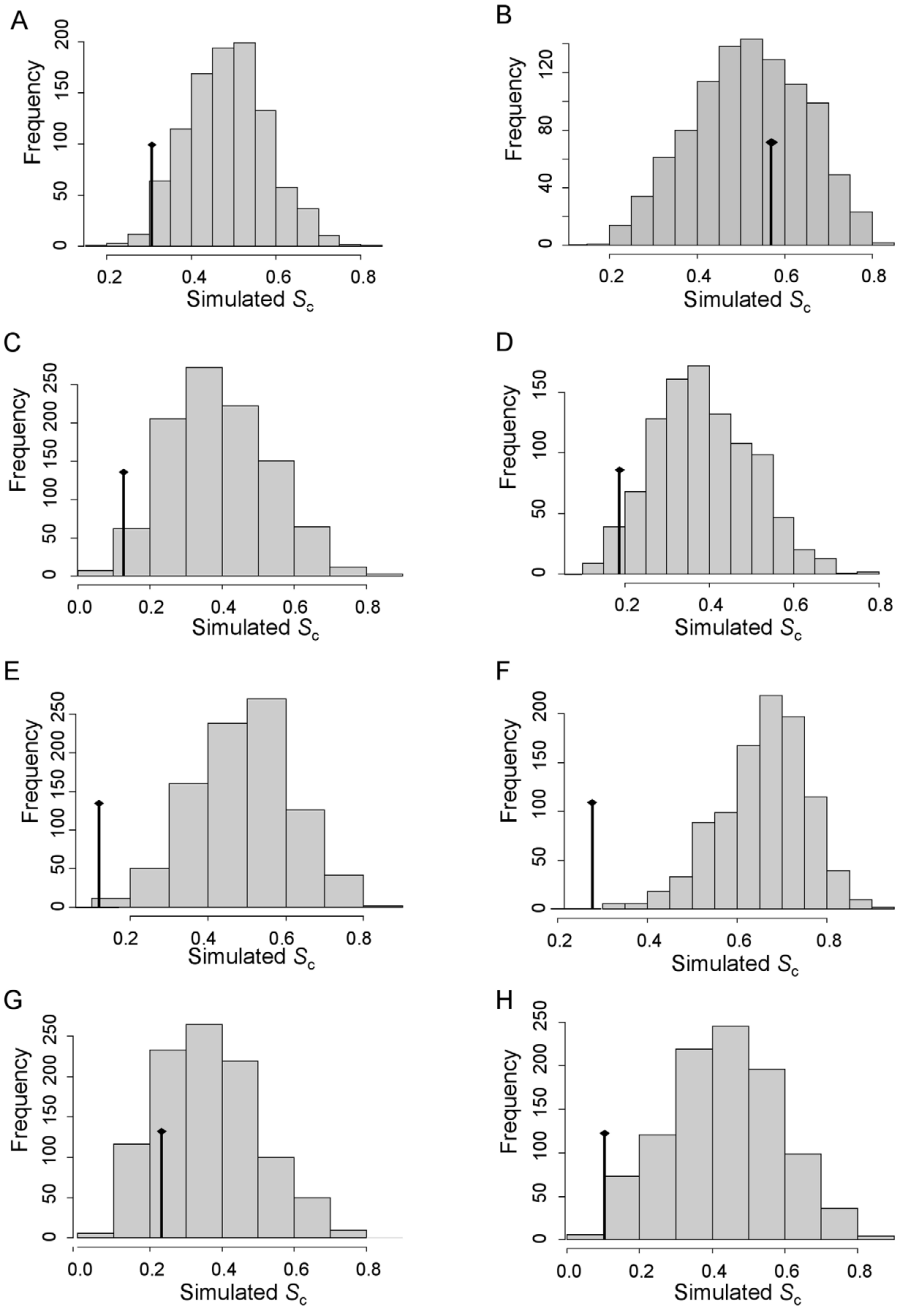
Theoretical distribution for *S*_c_ obtained from 1000 permutations. Diamonds show the observed values of *S*_c_. A: *dispersal fraction*: proportion of recaptures with inter-patch movement in multisite mark-recapture. B, C: respectively *Alpha1* and *alpha2* that describe the shape of a negative exponential dispersal kernel measured in small (<1.9 km) or large (>1.9 km) study sites. D: *P5km*, the probability of dispersal movement ≥5 km, estimated from the shape of inverse power dispersal kernels. E: *Daily moves*, the mean daily displacements in mark-release-recapture surveys. F, G, H: *FstL*, *FstR* and *FstC*, measures of the genetic structuring (*F*_ST_) from allozyme surveys respectively at the landscape scale (<100 km), the regional scale (100-600 km), or the continental scale (>600 km).

The visual examination of how dispersal diversity partitions onto phylogenetic trees shows that artificial nodes (within-species) generally bear a non-negligible part of the diversity in dispersal traits (Figure 2). The raw value of *S*_c_ represents the proportion of total trait diversity attributable to within-species variation, which here varies from 10% to 57% according to the dispersal metric considered (Table 2). This means that intra-species variability represented from 11% to 133% of the diversity in dispersal traits observed at the inter-specific level. For the majority of dispersal metrics, the artificial nodes accounted for a portion of the diversity that was not significantly different from a random distribution of trait diversity among the nodes of the corresponding tree, indicating that within-species variation in dispersal is not different from that observed at the inter-specific level (Table 2). Two out of eight observed *S*_c_ were significantly lower than the theoretical distribution obtained from permutations (*daily moves*, *FstL*: Table 2; Figure 3). *FstC* also showed a slightly significant trend to low variation within species. For the five other metrics, the variation observed between two populations of a species is of the same order of magnitude as that observed between two species, as shown by the position of *S*_c_ in the theoretical distribution (Table 2; Figure 3).

**Table 2:** Partitioning of the diversity in dispersal along the phylogeny of European butterflies: permutation tests (N = 1000 permutations).

Metric a	Statistic b	Hypothesis	Alternative	*P* c
*Dispersal fraction*	*S*_3_ = 0.417	Skewness to root	2-sided	0.076
	*S*_c_ = 0.307	Intra-specific conservation	Less	0.092
*Alpha1*	*S*_3_ = 0.578	Skewness to root	2-sided	0.583
	*S*_c_ = 0.570	Intra-specific conservation	Less	0.628
*Alpha2*	*S*_3_ = 0.319	Skewness to root	2-sided	0.014^d^
	*S*_c_ = 0.127	Intra-specific conservation	Less	0.092
*P5km*	*S*_3_ = 0.307	Skewness to root	2-sided	0.008^d^
	*S*_c_ = 0.188	Intra-specific conservation	Less	0.102
*Daily moves*	*S*_3_ = 0.381	Skewness to root	2-sided	0.036^d^
	*S*_c_ = 0.119	Intra-specific conservation	Less	0.015
*FstL*	*S*_3_ = 0.450	Skewness to root	2-sided	0.055
	*S*_c_ = 0.277	Intra-specific conservation	Less	0.008
*FstR*	*S*_3_ = 0.435	Skewness to root	2-sided	0.583
	*S*_c_ = 0.233	Intra-specific conservation	Less	0.384
*FstC*	*S*_3_ = 0.549	Skewness to root	2-sided	0.583
	*S*_c_ = 0.104	Intra-specific conservation	Less	0.054

a Replicates of a dispersal measurement for a given species are treated as if they were from virtual sister-taxa descending from an artificial terminal node in enlarged trees (see methods). Metrics are as in Table 1.

b Test S_3_ from Pavoine et al. [13]. S_c_ is the proportion of dispersal diversity attributed to within-species variability.

c P: P-values corrected for multiple comparisons.

d The diversity is significantly skewed towards nodes that were the most distant from tips in the original phylogeny (with 369 butterfly species considered).

Although artificial nodes and some other near-to-tips nodes stand for a significant part of the diversity in dispersal, this diversity generally remains significantly rooted into the phylogeny for most direct estimates of dispersal (Table 2: test *S*_3_). The diversity of indirect dispersal estimates (*F*_ST_) did not show a significant bias towards close-to-root nodes when accounting for within-species variability (Table 2: *S*_3_).

## Discussion

By partitioning the dispersal diversity along the phylogenetic trees, we considered the variation in dispersal traits observed at the species level in the light of that existing across related species. This method provides the first quantitative demonstration that, in European butterflies, dispersal is as diverse at the species level (among populations) as it is across species. However, for two direct estimators of dispersal, the variability in dispersal was significantly lower within-species than among-species, which indicates that trait conservation at the species level might also exist for some traits. This importance of within-species variation in dispersal traits will deeply impact the way dispersal models should be built to address specific questions such as the dynamics of metapopulations in fragmented landscapes or that of biological invasions. These implications are discussed below. We start here by some technical considerations about the method.

### Phylogenetic decomposition

Our method constitutes an original way to quantitatively appreciate the liability of functional traits in a phylogenetically explicit context. Here we used the decomposition of trait diversity to ask whether dispersal traits were less variable among populations of a species than across species in butterflies, but the method was constructed so that it could be applied to other questions and be extended to a suite of traits. For instance, by measuring the diversity at chosen nodes in the phylogeny, it is possible to detect regions in the phylogeny where a trait (or a combination of traits) shows a higher variability than random expectations. The null model in that case is that the trait diversity among the species that descend from that node is equivalent to the trait diversity expected by randomly drawing the same number of species from the species pool (that includes species at all tips of the phylogeny). Unfortunately, the data available did not allow us to make such analysis for dispersal in butterflies. For instance, some families were largely over-represented in our sample relatively to others (for instance, Nymphalidae represent 17% of the 369 European species, but are 20% to 53% of the species for which dispersal metrics were available: see Figure 2), which impeded us to test whether some families show high trait conservation whereas others are more labile regarding dispersal.

Analysing the partition of diversity for the combined facets of dispersal (that is: combining the dispersal propensity, the dispersal ability and the dispersal efficiency) was not feasible here because all these traits were available for different groups of species (see Figure 2). However, the statistic *S*_c_ could potentially be applied to a suite of functional traits as we measured trait diversity by the quadratic entropy index (see methods).

A complication of our approach comes from the use of published material. The studies from which we extracted the dispersal metrics did not all focus on dispersal. However, standardized Mark-Release-Recapture surveys allow to routinely detect among-patches movements (assimilated to dispersal), even when these are not central to the study; and genetic studies inform on the relative ability of populations to maintain gene flow through space, which is the net result of dispersal. However, we cannot rule out the possibility that part of the variation observed is attributable to the way dispersal was measured. For instance, the use of different sets of allozymes may result in slight differences in *F*_ST_, even within the same set of populations. In the same vein, there is a possibility that dispersal metrics were underestimated in some field studies. For instance, we have to assume that all possible sources for immigrants were properly surveyed, and that spatial scales were adequately chosen to detect most dispersal movements in the surveys that provided the dispersal metrics used here.

### Variability of dispersal among species

The dispersal propensity (here the *dispersal fraction*), the ability to disperse at given distances (*alphas*, *P5km*, *daily moves*) and the efficiency of dispersal movements (*F_ST_*) did not show the same pattern of diversity partitioning across butterfly species. Noticeably, only two metrics related to the ability to disperse (*alpha2*, *daily moves*) presented the signature of a phylogenetic signal while neither the dispersal propensity nor the dispersal efficiency did (Table 1).

Dispersal efficiency depends on several behavioural decisions of the butterfly: leaving its habitat, settling into another, and mating. On the contrary, we expect dispersal distances to be related to butterfly's flying capacity, which is related to morphological traits, like wing length or shape [14]. The heritability of morphological attributes is generally higher than that of behavioural traits [15], [16]. This difference may explain why the phylogenetic signal is only detected in dispersal ability and not in dispersal efficiency.

The absence of a phylogenetic signal on *F*_ST_ diversity might also be due to the fact that dispersal is not the only driver accounting for the spatial structuring of allozymic diversity, which is in effect the ultimate result of the contradicting forces of selection, random drift, mutation, and gene flow. All these forces probably vary among butterflies species, which may have confused the pattern of diversity in *F*_ST_. For instance, local adaptation is expected to occur with the selection of certain allozymes under certain sets of conditions in the environment [17], [18], [19], with the possibility of contrasting selective pressures on allozymes in different butterfly species. Moreover, gene flow itself may be not directly related to dispersal flows because it results from both dispersal movements and the relative ability of the disperser to transfer its genes to the next generation. The indirect relation between genetic structuring and dispersal flows might explain the absence of a detectable phylogenetic signal on *F*_ST_.

### Importance of within-species variability of dispersal

The phylogenetic perspective on dispersal variation shows that dispersal is highly variable at the species level. The importance of within-species diversity in dispersal traits was already suggested in our recent meta-analysis [20], but is here quantified for the first time. Only two out of the eight dispersal metrics considered tend to be conserved at the species level (Figures 2, 3; Table 2). The variation among different populations of the same species is generally not significantly less than the differences observed among species (like in the situation depicted right of Figure 1). *S*_c_ compares the diversity in dispersal at artificial nodes (that is the within-species diversity) to the diversity at all other nodes of the classification, and not only at other terminal nodes. This means that the difference in dispersal metrics between two populations of the same species could also have been observed between two randomly chosen species, not necessarily between particularly closely related species. This is a strong argument against dispersal as a species-specific, fixed trait.

The source for this high within-species variation is not investigated here, and is probably multiple. As mentioned, dispersal is condition- and phenotype-dependent, which may have caused variability in dispersal traits among populations of a species, either through the selection of contrasting dispersal patterns, or by the contrasting expression of butterflies' dispersal traits in different populations due to phenotypic plasticity or behavioural flexibility. Some evidence indicates that landscape configuration can cause within-species variation in dispersal propensity in butterflies [21], [22]. In a spider, Bonte et al. [23], [24] showed that contrasted landscape structures correlate with strong genetic variation in dispersal propensity. Others have shown that insect's performances related to dispersal ability, like flight endurance or the perceptual range (the distance at which the individual is able to perceive suitable habitats) are both heritable [25] and plastic [26]. Different traits associated to dispersal might thus either have been selected in population living in contrasted environments, or have been indirectly selected because they are dependent on morphological attributes selected for other reasons (indirect selection), or they might be expressed plastically by organisms experiencing contrasting conditions. To identify the relative importance of both processes (local adaptation *vs*. phenotypic plasticity) would help us to accurately predict how species will respond to spatial challenges like landscape fragmentation or climate change.

### Consistency of within-species variability

There is no general pattern in how the variability in dispersal is distributed among butterfly species: we did not reveal consistently ‘variable’ species and other ‘conservative’ species where all dispersal metrics were conserved. We showed that for a given species, the level of variation strongly depended on the metric considered (Figure 2). The studies from which data were extracted for our analyses were generally not designed so as to maximize the chance to detect differences in dispersal patterns, with the noticeable exception of *Proclossiana eunomia*, for which dispersal was measured in four landscapes along a gradient of fragmentation [21]. In other cases, study sites were chosen independently of a potential filtering on dispersal processes, which probably impeded the detection of a general pattern in species' variability in dispersal traits (if existing).

The heterogeneity of the variation observed among the metrics for a given species should be related to the heterogeneity of the dispersal process itself. What we call dispersal is in effect a process resulting from a suite of decisions, from emigration, through transfer, to immigration [27]. At first sight, our results suggest that those different dispersal estimates that we analyzed were under uncoupled selective pressures. In fact, the different dispersal metrics recorded in butterflies emphasize on a part of the whole dispersal process without taking into account the fitness rewards of the whole process. Complex feedbacks between them are possible; for instance, costs of transfer may limit the dispersal propensity [21]. How the various steps of the dispersal process co-vary or trade off with each other is still a relatively unaddressed question in dispersal research that certainly deserves further attention.

### Consequences for populations and species

Dispersal is a key process in the response of natural populations challenged by spatial problems such as the shift of suitable climatic envelopes [28] or the fragmentation of their habitats [29], and also participates in the propagation of alien species into new areas [30], [31]. We demonstrate here for the first time that dispersal is as variable between populations of a species as it is between species within a phylogenetically complex group. The accurate estimation of dispersal is therefore an essential prerequisite to realistically predict the demographic trajectories of threatened and invasive species with models. This estimation could be achieved either by measuring dispersal directly in the appropriate context, or by extrapolating from known causal relationships between context/phenotypes and dispersal abilities in the focal species. The identification of the processes at the origin of variation in dispersal traits should be addressed in future studies.

Because it participates to gene flow, dispersal is most probably not independent from other life-history traits. The few theoretical and empirical studies that investigated such relationships found strong dependency between dispersal and other traits [25], [32,32]. We show here that individuals from different populations of a given species vary in their propensity, their ability and their efficiency to disperse, which might cause local variation in the genetic conditions under which selection will operate. To identify and measure the dependency of life histories to dispersal is crucial to adequately predict the response of populations threatened by environmental changes, from both the demographic and the evolutionary points of view.

To conclude, the low conservation of dispersal traits we detected here within species will undoubtedly impact both the evolution and the metapopulation dynamics of butterflies, and hence must be accounted for in metapopulation modelling. This message is reinforced by the evidence that variability in metapopulation dynamics is dependent on both condition and phenotype [1], [34]. Considering dispersal as an invariant within species will severely limit our predictive capabilities for any spatial ecological problem. We show here that two metapopulations of a given species may differ in their dispersal abilities as much as do two metapopulations from different species. Predicting the fate of a metapopulation (and consequently that of a metacommunity) in a given region therefore requires that we estimate as exactly as possible the value of dispersal traits in the populations of interest. Accordingly, our results stress the need of incorporating dispersal variability into those predictive models that aim at forecasting species distribution according to global change. Dynamic modelling coupling habitat suitability models with spatially explicit stochastic (meta)population models including dispersal have been developed to explore factors that influence the viability of populations under stable and changing climate scenarios [35], [36]. However, our findings imply that single species-specific dispersal parameter should be replaced in such models by the use of a distribution of dispersal parameters sampled in each local population of interest.

## Methods

### Dispersal metrics

The various dispersal metrics published for European butterflies were recently reviewed by two of us [20]. We used here the dispersal metrics collected in this review that were available for several populations of the same species (Table 3). As we are interested in the within-species variation in dispersal, we considered only the dispersal metrics that were available for at least 10 species, among which at least three were represented by three or more replicates (i.e. dispersal estimated in at least three populations). Using these criteria, we selected eight metrics that reflect butterfly dispersal capability, coming from direct measurement in standardized Mark-Release-Recapture surveys (MRR) or, indirectly, from the genetic structure among populations inferred from allozyme screening.

**Table 3:** Butterfly dispersal data used for the phylogenetic partitioning of dispersal diversity.

Dispersal trait	Dispersal metric^a^	Number of species considered	Number of species with data available for >1 populations	Maximum number of populations/species for which the metric is available
Dispersal propensity	*Dispersal fraction*	25	11	6
Dispersal ability	*Alpha 1*	16	10	5
	*Alpha 2*	18	6	5
	*P5km*	28	10	6
	*Daily moves*	15	9	4
Dispersal efficiency	*FstL*	13	7	8
	*FstR*	15	3	6
	*FstC*	10	4	3

a Metrics are like in Table 1.

The relative dispersal propensity of butterflies was assessed by the *dispersal fraction*: the proportion of recaptured butterflies that were recaptured in a patch different from that of their first capture in MRR.

The relative dispersal ability of butterflies was described by four metrics, all coming from standardized MRR surveys. Butterflies' dispersal kernels–that is the inverse cumulative proportion of individuals moving certain distances–can generally be fitted either to a negative exponential or to an inverse power function. We used the shape of these two types of kernels as an indication of butterflies' dispersal ability. Negative exponential kernels were described by *alpha*, the only parameter of the function. As *alpha* was sensitive to the scale over which mark-recapture was performed, we considered separately alphas inferred from movement rates in study sites smaller or larger than the median length of the study sites. These were named respectively *alpha1* and *alpha2*. This grouping successfully eliminated the scale effect [20]. The shape of an inverse power kernel was summarized here by the estimated proportion of individuals moving five kilometres or more (*P5km*). The mean length of butterflies' daily moves (distance moved between successive captures) also can be used as a surrogate for dispersal ability. As this metric was scale-sensitive, we considered only *daily moves* measured in study sites larger than 0.7 kilometres: that is, in sites longer than the longest recorded mean daily move. This selection eliminated the scale effect [20] while keeping the required sample size.

Finally, indirect dispersal metrics inform on the relative efficiency of dispersal of butterflies. Although their sensitivity is questionable, *F*_ST_ is widely applied in population genetics and hence is widely available as indirect estimator of the relative dispersal ability of species. We considered here three spatial scales for *F*_ST_, which allowed to avoiding unwanted scale effects: *FstL*, *FstR* and *FstC* corresponded respectively to estimations of genetic structuring derived from the spatial structuring of allozyme diversity at the landscape scale (<100 km), the regional scale (100–600 km) or the continental scale (>600 km).

All eight dispersal metrics were Box-Cox transformed so as to conform to normality and were standardized before subsequent analyses.

### Phylogeny

The European butterflies' phylogenetic tree used is a combined tree constructed from published phylogenies of individual groups and, for groups with no phylogeny available, from formal classification into genera and subgenera [37]. This classification therefore has no branch lengths. Dispersal data were not available for all species in the tree (369), but for subsets of 10 to 28 species, according to the metric considered. We consequently pruned the phylogenetic tree to get eight distinct trees without missing dispersal data–one pruned tree for each metric considered.

When a given metric was available for several populations of a species, we considered those values as if they were from sister-taxa. To do that, we constructed eight enlarged trees, corresponding each to one of the pruned trees. In those trees, a terminal (artificial) node was added that supports the populations (now virtual sister-taxa) at the place where the species tip was in the pruned tree (see the eight enlarged trees in Figure 2). The phylogeny used has no branch length, which makes this reconstruction possible without making strong hypothesis about the length of the new branches (supporting populations and not species) relative to the other branches in the trees.

### Phylogenetic signal of dispersal diversity

To test the hypothesis that dispersal is constrained by phylogenetic relationships among European butterflies, we searched for a phylogenetic signal in the eight dispersal metrics by using Abouheif's statistic (*C_mean_*: [38]). We applied this statistic to pruned trees, where dispersal for each species was the mean observed values across its populations (wherever applicable). We applied the correction of Hochberg [39] on *P*-values to account for multiple tests.

### Partitioning dispersal diversity along the phylogeny

If the within-species variability in dispersal is negligibly low relative to the whole diversity observed across butterflies' species, we expect that (i) artificial nodes should not stand for a significant contribution in dispersal diversity as compared to other nodes, and (ii) the dispersal diversity would stay significantly rooted into the phylogenetic tree when accounting for the within-species variability.

To test this, we first applied the visual methodology proposed by Pavoine et al. [13], which calculates the contribution of each node in the phylogenetic classification to the diversity of a given trait–here each of the eight dispersal metrics–and gives a graphical representation of the trait diversity at different depths in the phylogenetic tree. Notice that this method ignores the ancestral values of the traits (the most probable value for the ancestral species represented by a given internal node) and is thus not influenced by the absolute value of the traits, but only by the variation in those values among the species present in the tree.

Next, to test if dispersal ability is conserved within a given species as compared to dispersal variation among species, we could not use classical statistical frameworks (including the ANOVA) because, for each dispersal traits, intra-specific trait values were available for a few species and a few populations within species only. Using ANOVA-like approach would have reduced the estimation of inter-specific trait variation to those species for which we also had estimations of intra-specific trait variation. Alternatively, we designed a permutation test, named trait conservation test, which was applied per dispersal trait. To do that, we measured trait diversity by the quadratic entropy index [40], [41], [42], which reduces to the variance if a single quantitative trait is considered as this is the case here. All trait values were standardized by standard deviation, so that the total variance was equal to 1. The contribution of a given internal node to trait diversity is equal to the diversity in trait values among the clades that descend from that node (*d_i_*) multiplied by the proportion of species that descend from that node (*p_i_*). The statistic used in that test (named *S*_c_ hereafter) is the sum of all *d_i_* * *p_i_* products over all artificial nodes (those nodes that connect the populations of a species and thus represent intra-specific variance). Because trait values were standardized, *S*_c_ is the proportion of trait variance attributable to intra-specific variation. *S*_c_ was computed on the observed values. Then we permuted the values of the trait across the tips of the extended phylogeny, which means that trait values are exchanged among all species and individuals within those species for which intra-specific values were available. This process thus mixed intra and inter-species variation. This permutation scheme impacts the *d_i_* values (trait diversities) leaving the *p_i_* values (species proportions) unchanged. Accordingly, our null model is that the diversity in trait values among the species that descend from an inter-specific node is equivalent to the diversity in trait values among the individuals that descend from an intra-specific node. We repeated this permutation process 1000 times. After each permutation, we computed the *S*_c_ statistic. This led to a theoretical distribution of *S*_c_ values corresponding to our null model. We then compared the observed value of *S*_c_ to those theoretical values. The intra-specific variation was considered lower than expected according to the null model if less than 5% of the theoretical values were lower or equal to the observed value of *S*_c_. Globally, we expect that the power of this test increases with the number of species and with the number of populations within species for which we have trait values. In that context, the statistic *S*_c_ has the advantage of attributing a higher weight in the analysis to those species with the highest number of populations considered. In case of within-species conservation of the trait, we expect that species' artificial nodes account for significantly less variability than random expectations (significant left-tailed *P*-value for *S*_c_). The test for trait conservation was here applied to each enlarged tree as a whole, contrasting artificial nodes (which bear the virtual sister-taxa representing the different populations of a species) to all other nodes, but it could possibly be applied separately to each artificial node, provided that enough data are available at the species level (which was not the case here). It could also be applied to a chosen node of the phylogeny, for instance to address specific questions of trait evolution. *P*-values were obtained from permutations, coded in R [43].

Finally, we used another permutation test, proposed by Pavoine et al. [13] to test if the values of the trait are organized within the phylogeny so that the diversity is clustered near the root of the classification: the skewness-to-root test, with statistic *S*_3_. We applied *S*_3_ to enlarged trees in order to see if diversity in dispersal traits remained significantly rooted within the tree when accounting for within-species variability. In order to avoid artefacts due to unbalanced pruning, and as the butterfly tree had no branch lengths, for this test, the nodes in enlarged trees were ordered according to the relative distance they had to the tips in the whole butterfly classification (with all 369 species).

For both *S*_c_ and *S*_3_, we applied the correction of Hochberg [39] for multiple tests.
